# Chronic Granulomatous Disease; fundamental stages in our understanding of CGD

**DOI:** 10.1186/1476-9433-5-4

**Published:** 2006-09-21

**Authors:** Tracy Assari

**Affiliations:** 1 Molecular Immunology Unit, The Institute of Child Health, University College London and Great Ormond Street Hospital for Children NHS Trust, 30 Guilford Street, London WC1N 3EH, UK

## Abstract

It has been 50 years since chronic granulomatous disease was first reported as a disease which fatally affected the ability of children to survive infections. Various milestone discoveries from the insufficient ability of patients' leucocytes to destroy microbial particles to the underlying genetic predispositions through which the disease is inherited have had important consequences. Longterm antibiotic prophylaxis has helped to fight infections associated with chronic granulomatous disease while the steady progress in bone marrow transplantation and the prospect of gene therapy are hailed as long awaited permanent treatment options. This review unearths the important findings by scientists that have led to our current understanding of the disease.

## Early CGD History

In 1954, at an annual meeting of the Society for Pediatric Research, Janeway and colleagues [1] first reported five cases of children with elevated serum gamma globulin levels that suffered from recurrent infections. At this time, the basis for their susceptibility was not identified. Three years later, four boys with hypergammaglobulinemia, suffering recurrent infections of the lungs, lymph nodes, and skin, with a presence of granulomatous lesions were described by Bridges *et al. *[2]. Landing and Shirkey [3] also described 2 boys with recurrent infection who had infiltration of visceral organs by pigmented lipid histiocytes. It was here that a new syndrome "fatal granulomatous disease of childhood" was first described. The disease was so called to depict the syndrome of recurrent infections whose sufferers invariably died in the first decade of life. Quie and colleagues further went on to demonstrate the defective bactericidal capacity of the polymorphonuclear leukocytes from affected males, thus meaning that these patients were unable to ward off infections [4]. Over the next ten years little more was known about the disease. Treatments using erythromycin and novobiocin antibiotics, along with regular surgical drainage, increased the survival rate of 4 years to one of 12 years. The use of the word 'fatal' was subsequently dropped from the clinical synonym as patient life expectancy increased with improved medicines and diagnostics, and was classified as the more commonly known chronic granulomatous disease (CGD).

## A genetic enzyme disorder

In the 1960s, studies on patient blood confirmed CGD to be a disease of impaired phagocytes. Holmes *et al. *[5] suggested that the cellular defect was 'a deficiency in one enzyme, either located within polymorph lysosomes or among those responsible for metabolic adaptations necessary for the normal function of the lysosomes or lysosomal enzymes'. This caused skepticism from some scientists who thought a deficiency of a single enzyme of this type was unlikely and that a less specific factor in the phagocytic process was responsible [6]. It was also thought that because the disease occurred in boys and was familial that the defect was X-linked. However, reports of the disease in females began to emerge, thereby revealing an autosomal recessive inheritance of the same phenotype [7,8]. It was then that Nathan and Baehner [9] showed that leukocytes from CGD patients, unlike normal human leukocytes, did not reduce nitroblue tetrazolium (NBT), a compound that converts to insoluble blue formazan product upon reduction by the superoxide anion (O2-^.^). This phenomenon was rapidly established as a sensitive clinical screening test for CGD that is still used today.

Over the next twenty years, over 150 scientific papers had been published reporting cases of patients with CGD describing their symptoms and similarities to other granuloma forming diseases. It was observed that CGD leucocytes could ingest micro-organisms but failed to kill the organisms responsible for the infections. Phagocytosis is normally accompanied by a marked increase in oxidative metabolism and studies had shown that NADPH oxidase was the respiratory enzyme responsible for bactericidal activity [10]. The hexose monophosphate shunt is responsible for generating reduced NADPH [10]. The critical deficiency in CGD cells is to generate O2-^. ^and other reactive oxygen species such as hydrogen peroxide (H_2_O_2_) [11]. The importance of H_2_O_2 _was illustrated by the fact that some bacterial species, such as *Streptococci*, that produces its own H_2_O_2_, could be killed by CGD leucocytes [12]. Following further corroboration that the deficiency in CGD was caused by a defect in the NADPH oxidase system in CGD patients, scientists began to research the formation of the free radical form of oxygen, O2-^.^, produced by NADPH oxidase during its respiratory burst, and showed that whereas normal leucocytes generated O2-^. ^during phagocytosis, CGD phagocytes were not able to do this [13-15].

Speculation that a b-type cytochrome may also be involved in this O2-^. ^generating activity began in 1979 from observations that a cytochrome-b associated with a particulate fraction of normal neutrophils, was absent from the neutrophils from some, but not all, patients with CGD [16]. Described as the heme-containing protein, cytochrome-b_558 _was proposed as a primary component of the microbicidal oxidase system of phagocytes. A multicenter European evaluation of its incidence and relevance was conducted in London [17] where it was found to be undetectable in all 19 of the men studied in whom the defect appeared to be located on the X chromosome. Thus cytochrome-b_558 _was hailed an important component of the microbicidal NADPH oxidase system and provided insight into its role in the enzyme complex. Borregaard and colleagues [18] demonstrated that approximately 90% of the cytochrome-b_558 _resides in the membrane of the specific granules of unstimulated human neutrophils and that the cytochrome-b_558 _translocates to the plasma membrane when the cells are stimulated. The authors speculated that the observed translocation was essential to the formation of an electron transport chain which generates O2-^.^, the single precursor from which all microbicidal oxidants ultimately arise.

## Unravelling the NADPH oxidase enzyme

The 1980s saw the formation of a disease-gene relationship. Linkage analysis using cloned, polymorphic DNA probes suggested a proximal location (Xp21) within CGD families [19]. Cytochrome-b_558 _was, so far, the only clearly defined component of this oxidase system and its absence provided the molecular basis of X-linked CGD, in which a profound predisposition to infection resulted from complete failure of this respiratory burst. Within a month of each other in March 1987, three separate groups working on the constituents of the phagocyte NADPH oxidase (phox) enzyme published their findings. Segal was the first to find that cytochrome-b_558 _had two subunits – a 23 K protein and the previously described 76–92 K glycoprotein. Reporting that the subunits were closely linked and remained associated with the heme of cytochrome-b_558_, neither protein was detected in the cells of five patients with X-linked CGD, whereas both were present in two with the autosomal recessive inheritance form of this disease. This was the first finding substantiating what we now know are the smaller and larger subunits of the phagocyte cytochrome-b_558 _heterodimer (p22phox and gp91phox respectively) [20]. Umei and colleagues then discovered a 66 kDa component [21] particulate in oxidase fractions obtained from patients with CGD, regardless of whether they contained cytochrome-b_558 _or not, and Curnutte and Scott [22] also found a soluble activation factor that was localized entirely to the cytosolic fraction with a mass of approximately 40 kDa. Later studies confirmed the exact molecular weight to be 47 kDa (p47phox).

In 1981, a variant or atypical X-linked form of CGD was described [23] whereby the gp91phox subunit was found in normal levels but only able to function partially. Curnutte [24] suggested that these rare type of CGD cases were allelic variants and patients with this uncommon cytochrome-b_558_-positive X-linked form of CGD have been reported by others since [25,26]. Today, classical X-linked CGD refers to cases where there is no respiratory burst activity demonstrable in a patient's neutrophils and the gp91phox subunit is absent. Autosomal recessive CGD is diagnosed when burst activity is abnormal and one of the other NADPH oxidase subunits is deficient. Variant or atypical CGD is diagnosed when a patient's neutrophils have demonstrable amounts of NADPH oxidase subunit present but oxidaitive burst activity is not at a level sufficient to fight off infection.

In 1986, the gp91phox subunit was first cloned [27], encoded from the CYBB gene, although little homology with other known protein sequences shed inadequate light on its function at the time. This was a huge advance in the molecular understanding of CGD and led to the development of animal models and the ability to determine what controls gp91phox function and activity; as well as being fundamental to current gene therapy development. Subsequently, by screening a promyelocytic leukemia cDNA library, Volpp *et al. *(1989) cloned and sequenced a cDNA encoding the 47-kD component of the NADPH oxidase system [28], Leto *et al. *(1990) [29] cloned a p67phox cDNA while Dinauer *et al. *[30] reported the structure of the gene for the 22-kD light chain of cytochrome-b_558 _and its chromosomal location, which served as a foundation for the analysis of genetic abnormalities at this locus in CGD. The cloning of p40phox by Wientjes *et al. *[31] provided important insights into its interactions with p67phox and p47phox in the cytosol. It was first suspected in the late 1980s that a GTPase might play a role in NADPH oxidase activation when it was demonstrated that guanine nucleotides were able to stimulate oxidase activity. The GTPase was subsequently identified and cloned as Rac1 or Rac2 and it is now clear that its presence is absolutely required for full oxidase function [32].

## Mutations in the X-linked and autosomal recessive forms of CGD

Research into CGD has improved our knowledge of the normal NADPH oxidase system. In some cases, the identification of specific mutations has provided insights at a molecular level. Most patients with CGD have mutations in the CYBB gene that encodes gp91phox, located at Xp21.1. Since the discovery of the CYBB gene no single mutation appeared to be responsible and a flood of research on mutations ensued, reporting large and small deletions, frameshifts and other mutations. The genetics of many hundreds of families with CGD were investigated and recounted and a mutation registry database for X-linked and autosomal recessive CGD was set up by Dirk Roos and MaunoVihinen from the University of Tampere in Finland [33] which presently lists 304 mutation entries from 267 unrelated families showing about 204 unique molecular events. Most of the X-linked mutations (174) are in the N-terminal domain. The number of mutations in other domains report: 50 in the FAD domain, 49 in the NADPH domain, 7 in the loop region, 3 in the upstream, the non-coding region before initiation codon, and 20 undefined gross deletions. The most common mutations are nonsense mutations (81), followed by missense mutations (78), frameshift deletions (42), intron mutations (34), frameshift insertions (30), and inframe deletions (6) [33,34].

Autosomal recessive CGD arises from mutations occurring in either the p67phox, p47phox or p21phox phagocyte oxidase proteins. Mutations in the NCF2 gene which encodes the p67phox represents about 5% of CGD cases whereas deficiencies of NCF1 which encodes p47phox represents about a quarter of all cases of CGD [35]. Unlike other CGD subtypes, in which there is great heterogeneity among mutations, 97% of affected alleles in patients with p47phox deficiency carry a characteristic 2-base pair deltaGT deletion in the NCF1 gene [36]. p22phox mutations are less common and 2 novel nonsense and missense mutations in the CYBA gene have been described [37]. Mutations in the corresponding genes responsible for the different genetic subgroups of CGD are shown in Table 1. Genetic defects in Rap1a, Rac, or p40phox havenot been reported in CGD [38], however, a dominant-negative mutation in the hematopoietic-specific Rac2 GTPase was identified in an infant with severe neutrophil dysfunction and a predisposition to bacterial infections similar to CGD and leukocyte adhesion deficiency [39].

**Table 1:** Classification of CGD subunits.

**CGD gene**	**Genetic Transmission**	**Frequency (%)**
gp91phox (CYBB)	X-linked	65
p47phox (NCF1)	Autosomal recessive	25
p67phox (NCF2)	Autosomal recessive	5
p22phox (CYBA)	Autosomal recessive	5

Mutations can occur in one of four NADPH oxidase subunits which give rise to X-linked or autosomal recessive CGD. The approximate frequencies of case occurrence are shown, with figures extrapolated from Clark *et al. *[35] and Dineaur *et al.*[38].

## Activation of the NADPH oxidase complex

The dormant oxidase consists of both cytosolic and membrane-bound components, but the active O2-^. ^generating complex is confined to the plasma membrane [1,2]. This implies that the cytosolic components must either act in a signaling capacity by modifying the membrane components, or that they must become directly associated with the membrane via a translocation process.

**Figure 1:**
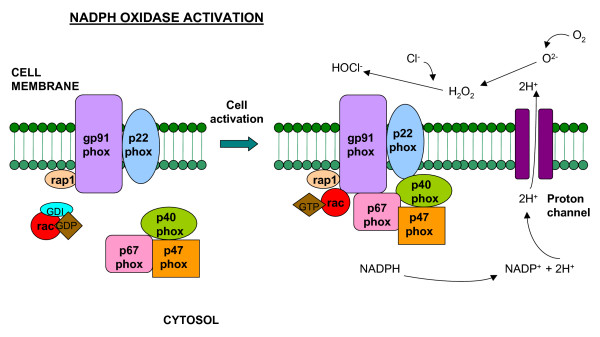
**Schematic representation of the NADPH oxidase enzyme**. The integral membrane of the phagocyte consists of two subunits: p22phox and gp91phox which respectively produce the smaller and larger chain of the cytochrome-b_558_. Two cytosolic subunits: p67phox and p47phox; a p40phox accessory protein and a Rac-GTP binding protein then translocate to the cell membrane upon cell activation to form the NADPH oxidase complex which generates a respiratory burst. Superoxide can react to form hydrogen peroxide and hypochlorus acid, which together participate in bacterial killing.

The 47 kDa phosphoprotein was found in both the cytosol and membranes after stimulation of neutrophils with phorbol myristate acetate and the phosphoprotein was detected in the cytosol preceding its detection in membranes [40]. Activation of the neutrophil oxidase system appeared to be dependent upon phosphorylation of the cytosolic 47 kDa protein [41], and incomplete phosphorylation was found to lead to failure of the subunit to translocate to the membrane for interaction with other membrane components of the oxidase. In intact neutrophils it was conclusively demonstrated that both p47phox and p67phox translocate to the membrane during activation of the respiratory burst [42]. This translocation was also normal in the variant X-linked form of CGD in which cytochrome-b_558 _is present but does not transfer electrons, demonstrating that the absence of translocation in other forms of CGD is not a secondary effect of the failure to generate O2-^. ^[43]. Ding and colleagues [44] were unable to detect any alterations in the protein kinases in CGD neutrophils that could explain these defects in phosphorylation of p47phox. The GTP-binding protein Rac2 was also found to migrate from the cytosol to the membrane cytoskeleton with p67phox and p47phox, indicating that Rac2 behaves like a dedicated component of the respiratory burst oxidase [45]. A schematic of NADPH oxidase activation is shown in Figure 1.

The primary biochemical defect in CGD that leads to impaired microbicidal activity was thought to be failure of phagocytes to generate sufficient quantities of reactive oxygen species (ROS), which are responsible for the so-called respiratory or oxidative burst. These compounds are derived from initial production of the extremely unstable and weakly bactericidal O2-^.^, which subsequently is dismutated into H_2_O_2_., In the presence of myloperoxidase which is delivered by primary granules into the phagosomes, H_2_O_2 _gives rise to more potent oxidants such as oxyhalides (most frequently hypochlorous acid), the hydroxyl radical (OH-^.^), or singlet oxygen (O^-.^) [46]. The high concentrations of ROS generated within the phagosome were thought to kill directly because of their oxidizing capacity.

The fact that phagocytes kill microbes through toxic oxygen radicals and their metabolites provided much of the biological basis for theories relating the toxicity of oxygen radicals to the pathogenesis of CGD. However, recent evidence [47] indicates that microbes might be killed by proteases, activated by the oxidase through the generation of a hypertonic, K^+^-rich and alkaline environment in the phagocytic vacuole. Thus, the role of ROS in the killing process is not as direct as previously thought, although, ROS are necessary to increase phagosomal osmolarity. Mice made deficient in neutrophil-granule proteases, but normal in respect of superoxide production and iodinating capacity, were unable to resist staphylococcal and candidal infections, suggesting that proteases are primarily responsible for the destruction of the bacteria [48]. Further research then showed that microbial killing and digestion were abolished when the BK_Ca _channel was blocked, revealing an essential and unexpected function for this K^+ ^channel in the microbicidal process [48].

## Defective inflammatory responses

In addition to recurrent life-threatening infections, patients often develop sterile granulomas in hollow organs, liver, lymphoid tissue and skin, without clinical evidence of infections [49]. The mechanisms involved in this aberrant inflammatory response are unknown. Inflammatory responses are finely balanced between pro-inflammatory and anti-inflammatory mediators and are important in generating an effective primary immune response and in clearing infection. Cultured cells from CGD patients have been shown to be deficient in their ability to produce anti-inflammatory mediators [50] and that neutrophils from these patients are defective in their ability to expose phosphatidylserine (PS), which is a recognition factor for phagocytic cells to clear apoptotic cell bodies. The externalized PS molecules on apoptotic cells, and subsequent internalization and degradation of apoptotic cells, is crucial to prevent the activation of an inflammatory response and persistence of inflammatory cells that do not undergo apoptosis and are not phagocytosed leads to an increase of necrosis and release of toxic granule contents that can cause chronic inflammation. Recently, we have shown that the rate of phagocytosis of apoptotic CGD neutrophils is also reduced (unpublished observations). It is not known why this occurs and further studies of the mechanisms of neutrophil apoptosis are necessary to understand the pathophysiology of its deficiency in CGD. Failure to successfully resolve inflammation can underlie the persistent inflammatory responses in CGD patients, as manifested by colitis, urinary tract obstruction, dysphagia, gastric outlet obstruction and chorioretinitis.

## Interferon-gamma therapy for CGD

In the late 1980s the potential of interferon gamma was investigated as a prophylactic therapeutic agent for CGD as preliminary studies in CGD patients demonstrated that brief in vitro or in vivo administration of recombinant IFN-γ (rIFN-γ) significantly enhanced phagocyte O2-^. ^production and *Staphylococcus aureus *bacterial killing. In two patients with variant X-linked CGD, rIFN-γ treatment in vivo was also associated with increased spectral levels of neutrophil cytochrome-b_558 _[51]. Subsequent studies found little evidence for transient increases in O2-^. ^production following rIFN-γ therapy, where O2-^. ^production was not sustained or associated with any change in cytochrome-b_558 _levels [52]. In some patients, rIFN-γ therapy was associated with the appearance of a small subset of circulating monocytes (1% to 20%) that were NBT-positive, suggesting that one possible mechanism by which rIFN-γ may benefit CGD patients was by partially correcting the respiratory burst defect in a subset of monocytes. Although it seemed that rIFN-γ therapy in the vast majority of CGD patients was not due to enhanced neutrophil NADPH oxidase activity, the mechanism of rIFN-γ action in CGD patients remains unknown. A study by Ahlin *et al. *[53] showed that rIFN-γ treatment of patients with CGD was associated with augmented production of nitric oxide by polymorphonuclear neutrophils. A comprehensive follow up study of 76 patients with CGD who received rIFN-γ found that its prolonged use in patients with CGD appeared to be safe and showed persistent reduction in the frequency of serious infection and mortality [54]. Recent studies looking at the cytochrome-b_558 _gene expression in Brazil [55] and Japan [56] found increased total messenger RNA (mRNA) levels in the CGD patients' cells suggesting that rIFN-γ improved mRNA splicing and concluded that rIFN-γ partially corrects a nuclear processing defect. These studies have lead to the consensus that only rare variants with splice site mutations can be improved with rIFN-γ therapy. rIFN-γ therapy is relatively expensive due to the high cost of recombinant human interferons, the large doses and lengthy course of administration necessary to achieve maximum response rates in recipients, and is not routinely administered prophylactically in Europe.

## A cure for CGD

Following the successful use of allogeneic bone marrow transplants to treat children with severe combined immune deficiency [57]. Goudemand and colleagues [58] made the first attempt to treat a case of CGD using bone marrow transplantation (BMT). Although in this case the transplant failed after two months due to tissue rejection recent advances in BMT expertise and technology have led to BMT becoming a successful treatment option for CGD patients. A recent review of cases revealed that 20 of 24 CGD patients are alive and disease free 1–7 years after transplant [59]. BMT can be successfully performed for CGD and remains an attractive option for children who have an HLA matched sibling donor and useful in selected very severe cases in which prophylactic therapy is problematic, although in many cases donors can be hard to find.

Gene therapy involves the permanent genetic correction of hematopoietic stem cells in which a vector is used to carry the corrective gene into the cells which are then re-introduced to the body. CGD is a particularly good candidate for gene therapy because low levels of functional phagocytes are expected to provide significant activity against pathogenic microbes. In 1992, Thrasher and colleagues [60] used cell lines from patients with defective p47phox as targets for reconstitution using retrovirus-mediated gene transfer, thus creating an in vitro model of gene therapy for this disease. This was shortly followed by retrovirus-mediated gene transfer of gp91phox cDNA from patients with X-linked CGD to reconstitute functional NADPH oxidase activity in B-cell lines [61]. The reconstitution of NADPH oxidase activity in cell lines from three unrelated patients, each of which had a different molecular defect in the gene, suggested that X-CGD would be a suitable disease for treatment by gene therapy. CGD mouse models have been developed by gene disruption, and preclinical studies on these animals using recombinant retroviral vectors have demonstrated reconstitution of functionally normal neutrophils and increased resistance to pathogens such as *Aspergillus fumigatus*, *Burkholderia cepacia *and *Staphylococcus aureus *[62]. These studies were extended to human cell lines, where retroviral vectors to restore NADPH oxidase activity was tested in human myeloid leukemic cell lines defective in superoxide production, as well as in primary CD34^+ ^cells obtained from X-CGD patients. It was shown that the level of O2-^. ^production in phagocytes derived from transduced cells was 69% of normal levels [63]. In the US, CGD gene therapy clinical trials targeted both the most common X-linked form of CGD as well as the autosomal recessive forms of this disease. Malech and colleagues [64] isolated hematopoietic stem cells from four X-CGD patients, infected the cells with a retrovirus containing the normal gp91phox gene, grew the transfected cells in culture, and infused them back into the original patients from which the cells were derived. Three of the four patients had sustained and continuous production of low levels of reconstituted neutrophils for 6 to 14 months. Of even greater interest, although still not understood, is the finding that two of the patients who had liver infections that resisted cure by conventional methods resolved during the course of treatment [64]. This suggests that the gene therapy approach could also be effective in treating CGD patients with severe intractable infections, although more targeted and longer term studies are needed.

Gene therapy trials for CGD in Europe began in 2004 through the collaborative efforts in Frankfurt and London. So far, two patients have been treated with retroviral vector and encouraging results show that both patients have significant levels of gene corrected cells and are clinically well (personal communication). Gene therapy holds great promise as an alternative treatment for patients without suitable marrow donors or where BMT is not a viable treatment option.

## Prospectives

It is estimated that one in 250,000 babies is born with CGD, although symptoms of the condition may not appear until after the first few months of age. Early reports documented the survival of patients beyond 7 years of age as 21% [65] which has since vastly improved due to advancement in treatments, and use of prophylactic antibacterials has seen a significant reduction in infectious complications. Over the last decade there has been momentous progress in scientific research into the underlying mechanisms of CGD. A recent search on the NIH Pubmed database showed over 2500 scientific publications on CGD dating back as far as 1959. The European CGD registry (funded by the CGD Research Trust) currently contains data on 490 CGD patients that provides a wealth of information for scientists, clinicians, nurses and patients alike. In the UK, the CGD Research Trust and Support Group is an active group who monitor and support CGD patients based in Britain, in addition to raising money and funding research into CGD [66]. Improved diagnosis ensures that patients with CGD are effectively managed and many people with CGD can carry on a normal life with few problems.

## Declaration of competing interests

The author(s) declare that they have no competing interests.
